# The *HMOX2* polymorphism contributes to the carotid body chemoreflex in European sea-level residents by regulating hypoxic ventilatory responses

**DOI:** 10.3389/fmed.2022.1000786

**Published:** 2022-11-03

**Authors:** Pierre Fabries, Catherine Drogou, Fabien Sauvet, Olivier Nespoulous, Marie-Claire Erkel, Vincent Marchandot, Walid Bouaziz, Benoît Lepetit, Anne-Pia Hamm-Hornez, Alexandra Malgoyre, Nathalie Koulmann, Danielle Gomez-Merino, Mounir Chennaoui

**Affiliations:** ^1^French Armed Forces Biomedical Research Institute – IRBA, Brétigny-sur-Orge, France; ^2^French Military Health Academy - Ecole du Val-de-Grâce, Paris, France; ^3^Laboratoire de Biologie de l'Exercice pour la Performance et la Santé – LBEPS – UMR, Université Paris-Saclay, IRBA, Evry-Courcouronnes, France; ^4^Vigilance Fatigue Sommeil et Santé Publique – VIFASOM – UPR 7330, Université de Paris Cité, Paris, France; ^5^41ème Antenne médicale, 5ème Centre médical des Armées, Dieuze, France; ^6^Aeromedical Center – CPEMPN – Percy Military Hospital, Clamart, France

**Keywords:** acute hypoxia, exercise, chemosensitivity, heme oxygenase-2, genetics, individual vulnerability, high altitude illness, Europeans

## Abstract

This study investigates whether a functional single nucleotide polymorphism of *HMOX2* (heme oxygenase-2) (rs4786504 T>C) is involved in individual chemosensitivity to acute hypoxia, as assessed by ventilatory responses, in European individuals. These responses were obtained at rest and during submaximal exercise, using a standardized and validated protocol for exposure to acute normobaric hypoxia. Carriers of the ancestral T allele (*n* = 44) have significantly lower resting and exercise hypoxic ventilatory responses than C/C homozygous carriers (*n* = 40). In the literature, a hypoxic ventilatory response threshold to exercise has been identified as an independent predictor of severe high altitude-illness (SHAI). Our study shows that carriers of the T allele have a higher risk of SHAI than carriers of the mutated C/C genotype. Secondarily, we were also interested in *COMT* (rs4680 G > A) polymorphism, which may be indirectly involved in the chemoreflex response through modulation of autonomic nervous system activity. Significant differences are present between *COMT* genotypes for oxygen saturation and ventilatory responses to hypoxia at rest. In conclusion, this study adds information on genetic factors involved in individual vulnerability to acute hypoxia and supports the critical role of the ≪ O_2_ sensor ≫ - heme oxygenase-2 - in the chemosensitivity of carotid bodies in Humans.

## Introduction

The ascent to high altitude exposes to decreased oxygen partial pressure due to the decrease of barometric pressure, inducing physiological responses such as increased ventilation and heart rate (HR), dedicated to maintain arterial oxygen saturation, blood pressure and homeostasis (1, 2). However, high altitude illness (HAI) intolerance can manifest as high altitude headaches, acute mountain sickness (AMS), high altitude cerebral edema (HACE) or high altitude pulmonary edema (HAPE) to varying degrees (3, 4). This depends on the altitude reached, the speed of ascent, the length of stay, and also intrinsic and genetic factors (1, 5). Thus, individual low chemoresponsiveness, implying decreased alveolar oxygen and increased hypoxemia, was found associated with acute high altitude intolerance (6–8). In case of long-term maladaptation, chronic mountain sickness can also be explained by maladjusted chemosensitivity (9, 10). Individual chemosensitivity to hypoxia can be assessed by the hypoxic exercise test, developed and validated by Prof. Richalet et al. (11), which measures the ventilatory response to poikilocapnic hypoxia during submaximal exercise (i.e., simulating the effort of walking to the summit of Mont Blanc) (11). The ventilatory response to exercise at sea-level involves different determinants (12). These include information from peripheral and central chemoreceptors, muscle demand and generated metabolic changes, as well as ventilatory fatigue, breathing patterns and changes in ventilation/perfusion ratios. In the Richalet's test, in healthy subject, the intensity and duration (30% maximal oxygen uptake, ~4 min) of exercise in hypoxia appear to be too low to modify the muscular metabolic balance, and to generate a ventilatory fatigue or to set up the hypocapnic inhibition mechanisms of hypoxic hyperventilation. The level of endurance training is one of the inputs influencing the responses to exercise in hypoxia, particularly in terms of oxygen saturation. The reproducible exercise test of Richalet et al. (11) allows the assessment of individual chemosensitivity to hypoxia through the calculation of exercise hypoxic ventilatory response (HVR) and hypoxic cardiac response (HCR), which are independent of altitude and exercise intensity (13). Low values of exercise HVR (<0.78 L/min/kg), low exercise HCR, and high values of changes in arterial oxygen saturation during exercise (DSaO_2_), were independent predictors of severe HAI (SHAI) (11). Clinical characteristics and trekking conditions are added to the measured physiological parameters to establish the score that detects subjects at high risk for severe high-altitude illness (14).

**Figure 1:**
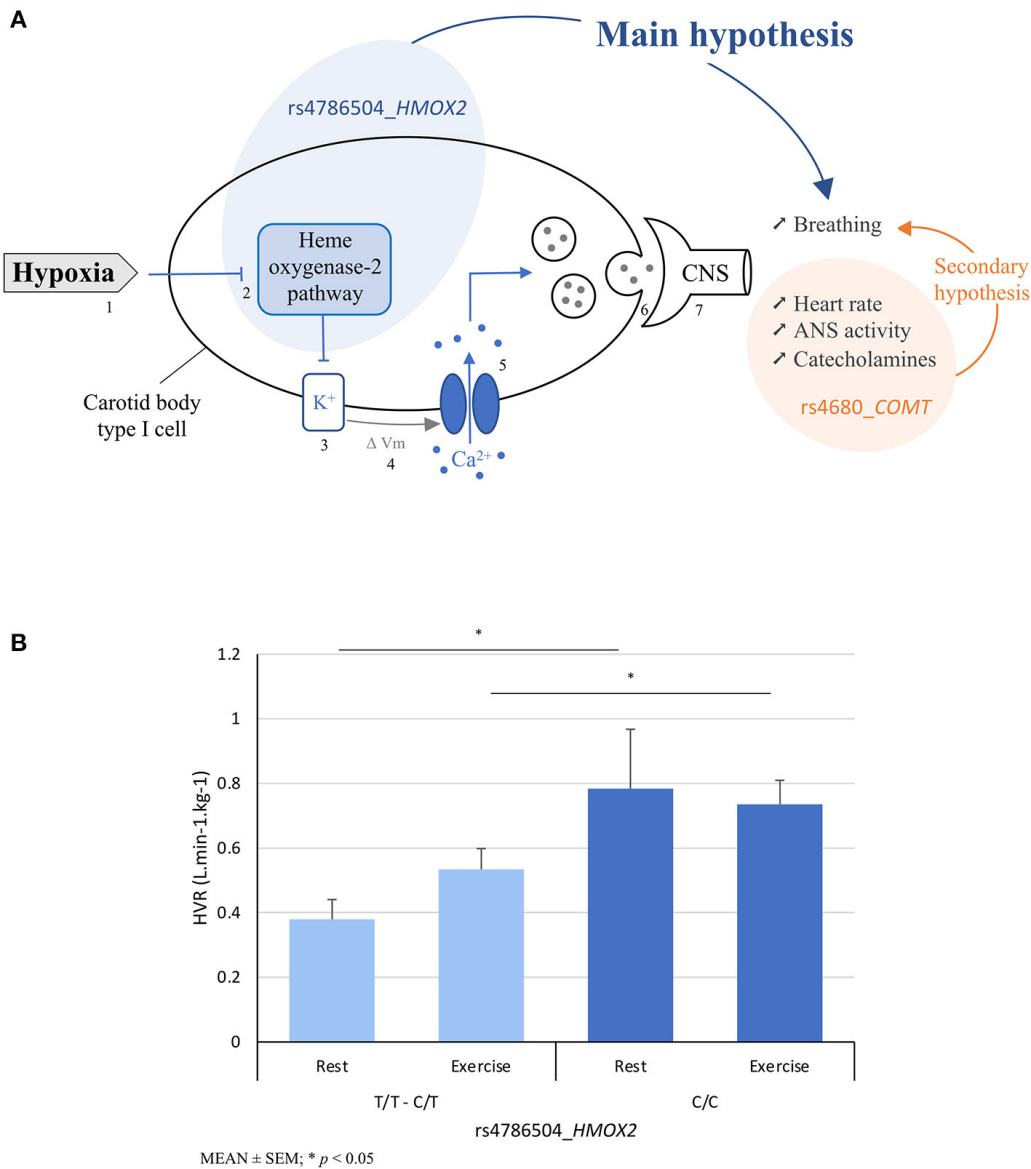
**(A)** Hypotheses using the schematic representation of the heme oxygenase-2 (HO-2) signaling in the detection of hypoxia by the carotid body: the main hypothesis is based on the association between rs4786504_*HMOX2* polymorphism and breathing (i.e., exercise hypoxic ventilatory response (HVR)) through HO-2 (blue circle, arrow), and the secondary hypothesis between rs4680_*COMT* and HVR through ANS activation and catecholamine release (orange circle, arrow); and **(B)** main finding regarding calculated resting and exercise hypoxic ventilatory responses as a function of rs4786504_*HMOX2* T>C. In the schematic representation, the different steps of the chemosensitivity are: (1) Hypoxia, (2) O_2_ sensing “chemosome”, (3) closure of potassium channels, (4) cellular depolarization, (5) opening of calcium channels and increase of cytosolic Ca^2+^ concentration, (6) neurotransmitters release and (7) information to central nervous system. ΔVm, membrane voltage change; ANS, autonomic nervous system; CNS, central nervous system.

Carotid body (CB) chemoreceptors located at the bifurcation of carotid arteries, and composed of type 1 and type 2 cells, are sensors of arterial O_2_, CO_2_, and pH (15). The type 1 glomus cells have a nerve connection and are called “sensor units”. They are the first step of the chemoreflex: an increase in nerve discharge is observed in hypoxia with a rapid increase in ventilation and HR through activation of the autonomic nervous system (ANS) (15). Several signaling pathways are thought to function as a “chemosome” within these cells to detect and mediate the response to hypoxia, with the predominant pathway being heme oxygenase (HO)-2, a carbon monoxide (CO)-producing enzyme considered as the primary O_2_ sensor (16, 17) (Figure 1A). The activity of this enzyme depends on the presence of O_2_ to degrade heme into biliverdin, Fe^2+^, and CO (18). Besides regulating plasma hemoglobin (Hb) concentration through heme degradation, HO-2 is involved in the hypoxic chemoreflex of carotid bodies by reducing CO production, which would lead to inhibition of potassium channels and then CB sensory nerve activity. HO-2 is a protein encoded by the *HMOX2* gene carried by chromosome 16. A study on *HMOX2* knockout mice thus showed a blunted hypoxic ventilatory response (19).

While more and more people go to high altitude for leisure or professional activities, few studies have explored the part of genetic factors involved in acute tolerance to hypoxia in Caucasians (20–22), and only one using the Richalet's test (23). In contrast, many studies have associated genetic polymorphisms with chronic adaptation in high-altitude populations, such as in the Andes and Tibet (2, 24). Most of the results concern intracellular responses downstream or upstream of the hypoxia inducible factor (HIF) (25–27). In 2010, Simonson et al. (28) identified *HMOX2* as one of the top HIF-independent candidate genes of high altitude adaptation in Tibetans. This was subsequently confirmed (29, 30). Tibetan population has thus acquired several key adaptive traits to cope with low oxygen conditions, such as increased ventilation at rest (31, 32), improved blood oxygen saturation levels, and lower Hb levels (33, 34). Recently, a functional *HMOX2* rs4786504 T>C polymorphism encoding the HO-2 protein was identified in relation to tolerance to high altitude adaptation in male Tibetans: carriers with the C allele at rs4786504 displayed lower Hb level as compared with the T allele carriers (35). These authors also demonstrated *in vitro* that the C allele could increase the expression of *HMOX2*, presumably leading to a more efficient degradation of heme which could contribute to maintaining a relatively lower Hb level at high altitude.

To our knowledge, no studies have examined the possible involvement of the gene coding for HO-2 in physiological responses to acute hypoxia. The objective of this study was to analyze the association between the rs4786504_*HMOX2* single nucleotide polymorphism (SNP) and chemosensitivity assessed by the ventilatory responses to the hypoxic exercise test (11) in an European sea-level residents population (Figure 1A). We also focused secondarily on the catechol-O-methyl transferase (*COMT*) SNP rs4680, which influences the enzymatic activity of COMT, a catecholamine-degrading enzyme. Admittedly, *COMT* is not considered a top candidate gene (compared with genes in the hypoxia-inducible factor (HIF) pathway that orchestrate the transcriptional response to hypoxia). We tested it secondarily in relation to autonomic nervous system (ANS) activation and its influence on increased HR and catecholamine release after detection of O_2_ depletion by glomus cells (36) (Figure 1A). The rs4680_*COMT* markedly affected enzyme activity, protein abundance, and protein stability (37). It is generally accepted that rs4680 is the main source of individual variation in human *COMT* activity (38) and it is associated with higher catecholamine response to exercise (39).

## Materials and methods

### Protocol

From 2019 to 2022, 84 healthy European sea-level residents under 45 years of age were included without a history of migraine or HAI, or high-altitude exposure in the past 3 months. We included subjects without sleep disturbances, verified by the Pittsburg sleep quality index <5 (40). Included women performed the protocol during the follicular phase of the menstrual cycle, because of physiological responses to hypoxic exercise depend on the ovarian cycle (41). The protocol was approved by the CPP SUD MEDITERRANEE III on 07 November 2019 (approval no.: 2019.10.03 bis _19.07.29.38738). All procedures were conducted in accordance with the Declaration of Helsinki and institutional protocols. All participants provided written informed consent before participation. Subjects were included at the French Armed Forces Biomedical Research Institute—IRBA—Brétigny-sur-Orge (Fr-91220) or at the Aeromedical Center—CPEMPN—Percy Military Hospital—Clamart (Fr-92140).

### Laboratory testing

In our study, the hypoxic ventilatory response at exercise was the main outcome. It was assessed through the test developed and validated by Professor Richalet (2012) and used extensively (42). It allows testing chemoreceptors to acute hypoxic stress and exploring peripheral chemosensitivity. During this test used in European centers for mountain medicine consultation (14), the subjects are exposed to the first four successive phases of 4 min of rest in normoxia, rest in normobaric hypoxia with a mask (FiO_2_ = 0.115 corresponding to an altitude of about 4800 m), submaximal exercise in hypoxia (about 30% of maximal aerobic power which equals to 40–50% of HR reserve based on the value of theoretical maximal HR) and submaximal exercise in normoxia at the same intensity. We did not carry out the 5th phase in normoxia which allows to obtain the decrease in power output. Room air temperature was maintained at 22°C. The normobaric hypoxia was obtained by using a mixture of gases in the mask (Altitrainer, SMTEC ^®^). Antibacterial filters for spirometers were used for all subjects. The exercise was performed on an electrically braked cycloergometer (Excalibur, Lode ^®^). Minute ventilation (L.min^−1^), HR (bpm) and arterial oxygen saturation (SaO_2_) (%) were measured with a Quark CPET, Cosmed ^®^ device and a Nonin ^®^ sensor at the level of the earlobe previously vasodilated (Dolpic, PharmUp ^®^). Systolic and diastolic blood pressure (SBP–DBP) were measured at each phase (Tango M2, SunTech ^®^). Hypoxic ventilatory and cardiac responses (HVR and HCR) and desaturation (DSaO_2_) were calculated, respectively at exercise (e) and at rest (r), using the mean values of minute ventilation ($\overset{\cdot }{V}$E, L/min), HR and SaO_2_ during the last minute of the different phases:

Exercise HVR = $\text{Δ}\overset{\cdot }{V}$E_e_ / DSaO_2e_/Body weight^*^100 (L.min-1.kg-1)

Resting HVR = $\text{Δ}\overset{\cdot }{V}$E_r_ / DSaO_2r_/Body weight^*^100 (L.min-1.kg-1)

Exercise HCR = ΔHR_e_ / DSaO_2e_ (min-1.%-1)

Resting HCR = ΔHR_r_ / DSaO_2r_ (min-1.%-1)

DSaO_2_ at exercise = ΔSaO_2e_ (%)

DSaO_2_ at rest = ΔSaO_2r_ (%)

Among these criteria, the ventilatory response to hypoxic exercise (exercise HVR) has been shown to be the best predictor of poor tolerance to high altitude with a threshold fixed <0.78 L.min-1.kg-1. These results also allow the calculation of a clinicophysiological score for the detection of subjects with high risk for severe high-altitude illness (a score > 5.5). The score included history of SHAI, rapid ascent, geographical location, female sex, regular endurance physical activity and exercise HVR, exercise HCR and DSaO_2_ at exercise (11, 14, 43).

### Sample collection, biology and genotyping

A total of 10 mL of blood was collected from the antecubital vein and stored in our laboratory for analysis. An automated system (XN-L, Sysmex ^®^) was used to determine the plasma Hb concentration (g/dL) and the hematocrit level (%).

Determinations of rs4786504_*HMOX2* and rs4680_*COMT* polymorphisms were performed at IRBA using LAMP-MC technology. LAMP-MC consists in a lysis of cells from whole blood followed by the amplification of the target sequence at a constant temperature around 65 C using simultaneously three sets of primers, a polymerase with high strand displacement activity in addition to a replication activity and a fluorophore-labeled probe (44). Detection of homozygous wild, heterozygous and homozygous mutant genotypes is performed by melting curve analysis after amplification. LAMP-MC assays were realized by use of the customized *HMOX2* and *COMT* kit (LaCAR MDX, Liège, Belgium). A positive control and a negative control were supplied. Samples, controls and reagents were thawed at room temperature and the reactions were set up with 5 μL blood or control, or one swab, in 1 mL of lysis buffer. After 10 min of incubation at room temperature, 5 μL of the lysed sample or control were added to 20 μL reaction buffer per well. The SNP was detected by a specific reaction buffer, containing all reagents specific for the amplification and detection. The 8 wells strip, containing 6 unknown samples and 2 controls, was briefly centrifuged and then placed in the analyzer (LC-Genie IIITM v3.17). Following the 40 min amplification step at 65 C, the mix were cooled down to 35 C to allow fluorophore-labeled probe annealing. Then melting curves were generated in the temperature range of 35–80 C with ramp rate of 0.2 C/s. The increasing of temperature induces the separation of the fluorophore from the quencher and the generation of a fluorescence signal. According to the design of each SNP probe, the presence or the absence of mutation decreases the dehybridization temperature of probe.

### Statistics

For descriptive purposes, we calculated genotype frequencies for each individual polymorphism. To test genotype-phenotype associations, we considered measured and calculated physiological outcomes (at rest and during exercise, in normoxia and hypoxia), and biological outcomes (hemoglobin and hematocrit). For the association with *HMOX2* (two modalities, T/T–C/T vs. C/C genotypes), we used the student's *t*-test (*n* > 30) or Welch's *t-*test (depending on the homogeneity of variance assessed by the Levene test). For association with *COMT* (three modalities, G/G, G/A, A/A genotypes), we previously check the normality assumption using a Shapiro-Wilk test, and we used a one-way ANOVA followed by a Tukey *post-hoc* test to identify differences between genotypes. In addition, clinical characteristics, endurance training levels, and hemodynamic parameters are presented to highlight the homogeneity of the study sample. We performed a Chi-square analysis and calculated odds ratios with their 95% confidence interval (IC 95) to test the relationship between the *HMOX2* polymorphism and physiological phenotypes considering validated thresholds of exercise HVR, exercise HCR, exercise DSaO_2_, plus Richalet's score value. To test the association between the calculated Richalet score and hematological parameters, we used the student *t*-test (*n* < 30) after testing the normality of their distribution (Shapiro-Wilk test), or the Welch's *t*-test (depending on the homogeneity of variance assessed by the Levene test). Clinical characteristics were also tested to highlight the homogeneity of the two subgroups in the Richalet's score. In order to assess the association between *HMOX2* and *COMT* polymorphisms on the calculated parameters HVR, HCR, and DSaO_2_ at rest and during exercise, we used a two-way ANOVA (*HMOX2* and *COMT* fixed levels). Because the number of subjects with the T/T genotype was low (*n* = 9), we analyzed the homozygous C/C mutated carriers of the *HMOX2* polymorphism vs. the carriers of the T allele (C/T + T/T), in order to respect the conditions for the application of statistical tests, in particular the chi-square. The statistical software Jamovi ^®^ version 1.6.23.0 was used.

## Results

### Association between rs4786504_*HMOX2* and physiological responses during hypoxic exercise testing

The allele frequency for allele of rs4786504_*HMOX2* is given in Table 1. The genotype prevalence of this SNP is similar to the 1000 Genomes Project data for European population on the GRCh38 reference assembly (https://www.internationalgenome.org, last access the 15/04/2022). The clinical and physiological characteristics of the 84 included subjects according to the rs4786504 genotypes are given in Table 2. The two groups (C/T–T/T, C/C) are similar in gender distribution, age, height, body weight, body mass index (BMI), endurance training, hemodynamics parameters (SBP and DBP), Hb and hematocrit levels. They are similar in measured oxygen saturation, HR and ventilation during normoxia and normobaric hypoxia, at rest and during submaximal exercise. There is a significant relationship between the *HMOX2* SNP and calculated hypoxic ventilatory responses (HVR) at rest and submaximal exercise. Compared to T allele carriers, C/C mutated homozygous have higher ventilatory responses to hypoxia at rest (*p* = 0.042) and at submaximal exercise (*p* = 0.043) (Figure 1B). The individual values of ventilatory responses to hypoxia are presented in Supplementary Figures 1A,B. There are no relationships between the SNP and differences in oxygen saturation (DSaO_2_) or calculated hypoxic cardiac responses (HCR) nor at rest nor at exercise. However, a tendency is observed in the HCR at rest, which seems to appear higher for mutated homozygous C/C than for T allele carriers (*p* = 0.134).

**Table 1:** Genetic distribution of *HMOX2* and *COMT* polymorphisms in our population and in the data base of 1000 genomes.

**Genetic polymorphisms (chromosome: location)**	**Genotypes**	***n* (%, [IC 95])**	**1000 Genomes – European** **(%)**	**Age (years)**	**Male (%)**
rs4786504_*HMOX2* (16:4537693)	T/T (ancestral) - C/T	44 (52.4 [41; 63])	49.5	27.7 ± 6.44	84.1
	C/C	40 (47.6 [37; 59])	50.5	26.9 ± 6.96	87.5
rs4680_*COMT* (22:19.963.748)	G/G (ancestral)	30 (35.7 [26; 47])	32.9	26.5 ± 6.36	86.6
	A/G	36 (42.9 [32; 54])	47.1	27.8 ± 7.52	83.3
	A/A	18 (21.4 [13; 32])	26.4	27.7 ± 5.46	88.8

Mean ± SD.

**Table 2:** Clinical, biological and physiological characteristics of subjects according to *HMOX2* and *COMT* polymorphisms.

	**rs4786504_** ***HMOX2***		***p***	**rs4680_** ***COMT***			***p***	**Total population**
	**C/T - T/T**	**C/C**		**G/G**	**G/A**	**A/A**		
	***n* = 44**	***n* = 40**		***n* = 30**	***n* = 36**	***n* = 18**		***n* = 84**
**Clinical characteristics**								
Male (%)	37 (84.1)	35 (87.5)	0.656	26 (86.7)	30 (83.3)	16 (88.8)	0.845	72 (85.7)
Age (years)	27.7 ± 6.4	26.9 ± 7	0.561	26.5 ± 16.4	27.8 ± 7.5	27.7 ± 5.5	0.71	27.3 ± 6.7
Height (cm)	178 ± 8	176 ± 7	0.455	178 ± 7	177 ± 9	177 ± 8	0.948	177 ± 8
Weight (kg)	74.1 ± 11.3	73.2 ± 9.5	0.694	72.8 ± 12.1	74 ± 9	74.3 ± 10.7	0.879	73.6 ± 10.4
BMI	23.4 ± 2.8	23.5 ± 2.6	0.868	23.1 ± 3	23.7 ± 2.7	23.5 ± 2.1	0.659	23.5 ± 2.7
Endurance (%)	50	45	0.737	46.7	44.4	55.5	0.737	47.6
SBP at rest normoxia (mmHg)	124.4 ± 12.3	128.2 ± 14	0.185	124.7 ± 15.2	125.1 ± 12.5	131.1± 10.2	0.126	126 ± 13.2
DBP at rest normoxia (mmHg)	76.8 ± 8.7	76.5 ± 10.7	0.898	75.9 ± 10	77 ± 1.5	77.2 ± 11.1	0.889	76.6 ± 9.7
**Biological characteristics**								
Hemoglobin (g/dL)	14.8 ± 1.1	15 ± 1	0.373	15 ± 1.2	14.7 ± 0.9	15 ± 1.2	0.582	14.9 ± 1.1
Hematocrit (%)	42.7 ± 3.6	43.1 ± 2.8	0.579	43.4 ± 3.5	42.8 ± 2.6	42.4 ± 3.8	0.596	42.9 ± 3.2
**Physiological characteristics**								
**SaO**_**2**_ **(%)**								
Rest (normoxia)	98.7 ± 0.5	98.7 ± 0.5	0.98	98.8 ± 0.5	98.7 ± 0.4	98.8 ± 0.5	0.527	98.7 ± 0.5
Rest (hypoxia)	88.8 ± 4.5	88.7 ± 4.8	0.898	**88.6** **±4.4**	**87.6** **±4.5**	**91.2** **±4.3** *	**0.024**	88.7 ± 4.6
Exercise (normoxia)	97.9 ± 0.8	97.6 ± 1	0.231	97.9 ± 0.8	97.5 ± 1	98 ± 0.9	0.108	97.8 ± 9
Exercise (hypoxia)	76.9 ± 6.9	76.4 ± 6.1	0.723	77.3 ± 5.8	75.8 ± 7.2	77.3 ± 6.2	0.611	76.7 ± 6.5
**Heart rate (min-1)**								
Rest (normoxia)	79.5 ± 14.6	77.8 ± 14.6	0.615	80.5 ± 13.6	75.4 ± 14.2	82.3 ± 16.1	0.201	78.7 ± 14.5
Rest (hypoxia)	90.9 ± 16.5	91.5 ± 17.1	0.869	93.1 ± 15.7	88.2 ± 16.4	93.8 ± 18.7	0.383	91.2 ± 16.7
Exercise (normoxia)	100 ± 11.3	103 ± 12.1	0.27	103 ± 12.7	100 ± 10.9	102 ± 11.9	0.617	101 ± 11.7
Exercise (hypoxia)	120 ± 12.8	124 ± 12.3	0.237	123 ± 12.8	120 ± 11.9	124 ± 13.7	0.377	122 ± 12.6
**Ventilation (L.min-1)**								
Rest (normoxia)	10.7 ± 3.6	10.3 ± 3.7	0.62	10.6 ± 3.6	10.5 ± 3.5	10.5 ± 4.1	0.998	10.5 ± 3.6
Rest (hypoxia)	13.2 ± 4.4	13.8 ±4.3	0.527	13.7 ± 4.2	12.9 ± 3.7	14.2 ± 5.7	0.598	13.5 ± 4.3
Exercise (normoxia)	21.5 ± 5.8	21.5 ± 5.6	0.957	21.2 ± 5.8	20.8 ± 4.2	23.5 ± 7.8	0.409	21.5 ± 5.7
Exercise (hypoxia)	29.4 ± 10.2	31.8 ± 8.2	0.245	30.5 ± 8.7	29.3 ± 6.7	32.9 ± 14	0.547	30.5 ± 9.3
Power output at exercise (W)	45.8 ± 22.9	45 ± 26.9	0.874	43.5 ± 27	45.9 ± 21.9	47.8 ± 27.5	0.864	45.4 ± 24.8
**Calculated parameters**								
DSaO_2_ at rest (%)	9.9 ± 4.3	10.1 ± 4.8	0.897	**10.2** **±4.3**	**11** **±4.4**	**7.6** **±4.2** *	**0.027**	10 ± 4.5
DSaO_2_ at exercise (%)	21 ± 6.6	21.2 ± 5.9	0.852	20.6 ± 5.7	21.7 ± 6.9	20.7 ± 6	0.778	21.1 ± 6.2
Resting HCR (min-1.%-1)	1.3 ± 0.7	1.6 ± 1.1	0.134	1.3 ± 0.8	1.3 ± 1	1.8 ± 1	0.156	1.4 ± 0.9
Exercise HCR (min-1.%-1)	1 ± 0.3	1 ± 0.3	0.777	1 ± 0.2	0.9 ± 0.3	1.1 ± 0.4	0.3	1 ± 0.3
Resting HVR (L.min-1.kg-1)	**0.4** **±0.4**	**0.8** **±1.2**	**0.042**	**0.6** **±1.1**	**0.3** **±0.2**	**1** **±1.1** *	**0.031**	0.5 ± 0.4
Exercise HVR (L.min-1.kg-1)	**0.5** **±0.4**	**0.7** **±0.5**	**0.043**	0.7 ± 0.5	0.5 ± 0.3	0.7 ± 0.6	0.41	0.6 ± 0.4

Mean ± SD.

* *p* < 0.05 *COMT* A/A vs. G/A.

The data for the whole population are in the last column on the right. BMI, body mass index; SBP, systolic blood pressure; DBP, diastolic blood pressure; SaO_2_, oxygen saturation; DSaO_2_, difference on saturation; HCR, hypoxic cardiac response; HVR, hypoxic ventilatory response.

In bold values for respective genotypes and their significant differences.

The results of the chi-square analysis between *HMOX2* genotypes and calculated parameters are given in Table 3. Sixty-two percent of subjects with low exercise HVR (<0.78 L.min-1.kg-1) are T allele carriers. In contrast, only 24% of subjects with a high exercise HVR carried T allele (Chi-square = 9.16; *p* = 0.002; OR = 5.2 [1.69; 16.03]). No correlation is found between genotypes and exercise HCR, DSaO_2_ at exercise and Richalet's score (chi-square < 3.84).

**Table 3:** Contingency table showing the correlation between *HMOX2* genotypes and calculated parameters; HVR, hypoxic ventilatory response; HCR, hypoxic cardiac response; DSaO_2_, difference on saturation.

			***HMOX2***		**Chi-square**	***p***	**OR [IC 95]**
		**N**	**T/T - C/T**	**C/C**			
**Exercise HVR**	**≥0.78 L.min-1.kg-1**	**21**	**24%**	**76%**			
	**<** **0.78 L.min-1.kg-1**	**63**	**62%**	**38%**	**9.16**	**0.002**	**5.2 [1.69; 16.03]**
Exercise HCR	≥ 0.84 min-1.%-1	56	52%	48%			
	< 0.84 min-1.%-1	28	54%	46%	0.02	0.8	1 [0.43; 2.66]
DSaO_2_ at exercise	< 22 %	41	60%	40%			
	≥ 22 %	43	44%	56%	2.31	0.1	0.5 [0.21; 1.22]
Richalet's score	≤ 5.5	65	52 %	48 %			
	> 5.5	19	52 %	48 %	0	0.98	0.99 [0.36; 2.76]

In bold values for respective genotypes and their significant differences.

### Association between rs4680_*COMT* and physiological responses during hypoxic exercise testing

The allele frequency for allele rs4680_*COMT* is given in Table 1. The three genotype groups (G/G, G/A, A/A) are similar in gender distribution, age, height, body weight, BMI, endurance training, hemodynamics parameters (SBP and DBP), Hb and hematocrit levels. They are also similar in measured HR and ventilation during normoxia and hypoxia at rest and during submaximal exercise. There is a significant association between polymorphism and resting HVR. The A/A (also named Met/Met) mutated homozygous have a higher response than ancestral homozygous G/G (also named Val/Val) and heterozygous G/A (*p* = 0.031). The individual values of ventilatory responses to hypoxia are presented in Supplementary Figures 1C,D. The A/A mutated homozygous also have lower DSaO_2_ at rest than heterozygous and G/G (*p* = 0.027). Measured oxygen saturation in resting hypoxia of A/A homozygous is higher than the other two groups (*p* = 0.024). Regarding the HCR, there is a tendency between the response at rest. The A/A mutated response seems to be higher than G/A and G/G (*p* = 0.156).

Furthermore, there is no significant interaction between the *HMOX2* and *COMT* polymorphisms on the calculated parameters HVR, HCR, and DSaO_2_ at rest and during exercise (*p* > 0.05).

### Hematological parameters and score for the detection of subjects with high risk for severe high-altitude illness

The calculated clinicophysiological score for the detection of subjects at high risk of severe high-altitude illness is presented in Table 4. The two groups of subjects (a score of ≤ 5.5 and > 5.5, respectively) are similar in gender distribution, age, height, body weight and BMI. Hb level is significantly lower in the group with a score > 5.5 (*p* = 0.007), as well as the hematocrit level (*p* = 0.039) compared to the group with a score below 5.5.

**Table 4:** Clinical and biological characteristics according to the Richalet's score; BMI, body mass index.

	**Richalet's score**		**Total**	***p***
	** ≤ 5.5**	**> 5.5**		
	***n* = 65 (77.4%)**	***n* = 19 (22.6%)**	***n =* 84 (100%)**	
Male (%)	58 (89.2)	14 (73.7)	72 (85.7)	0.131
Age (years)	27.3 ± 6.6	27.2 ± 7.1	27.3 ± 6.7	0.949
Height (cm)	177 ± 8	176 ± 8	177 ± 8	0.542
Weight (kg)	73.6 ± 10.1	73.9 ± 11.6	73.6 ± 10.4	0.896
BMI	23.3 ± 2.5	23.8 ± 3.2	23.5 ± 2.7	0.494
**Hemoglobin (g/dL)**	**15** **±1.1**	**14.4** **±0.7**	**14.9** **±1.1**	**0.007**
**Hematocrit (%)**	**43.3** **±3.3**	**41.6** **±2.5**	**42.9** **±3.2**	**0.039**

MEAN ± SD. In bold values for respective Richalet's score and their significant differences.

## Discussion

This is the first study in a relatively large group of European sea-level residents to associate the *HMOX2* polymorphism with hypoxic ventilatory responses at exercise and rest in the acute hypoxia functional test that assesses individual chemosensitivity. Secondarily, the *COMT* polymorphism, an enzyme involved in catecholamines degradation, is also associated with responses at rest (i.e., resting HVR; DSaO2 and SaO2 at rest).

The rs4786504_*HMOX2* polymorphism appears to be directly involved in chemosensitivity at the glomus cell level by modulating ventilatory responses to hypoxia, and HO-2 is considered the “O_2_ sensor” in the “chemosome” of type 1 glomus cells of carotid bodies (Figure 1A) (16, 17). The rs4786504_*HMOX2* has been shown *in vitro* to be associated with variable *HMOX2* regulatory activity, with that of the C allele being significantly higher than that of the T allele (35). This is likely because the T allele have just one Sp1 binding instead of 2 in the tata box gene regulatory region (35).

Our results show that homozygous C/C carriers, with higher expression of *HMOX2* (35), have higher submaximal exercise and resting HVR than T allele carriers (Figure 1B). The distribution of allelic frequencies in our sample is consistent with the expected data from the 1000 Genomes database in Europeans. Exercise-induced HVR is presented as the best independent predictor of the occurrence of SHAI (severe AMS, HACE, HAPE) with a defined threshold when exercise-induced HVR is low <0.78 L.min-1.kg-1. In our study, C/C carriers reach this value at rest and submaximal exercise does not affect the response (*p* = 0.806). Further studies would be interesting on rs4786504_*HMOX2* in C/C carriers with higher intensity exercise during hypoxia to increase the exercise HVR above the baseline value of 0.78 L.min-1.kg-1. We also showed that C/C carriers are at lower risk of SHAI than T allele carriers (Chi-square = 9.16; OR = 5.2 [1.69; 16.03]). Our results are in line with studies showing blunted HVR in knock-out animals *HMOX2*–/– (19), and increased resting ventilation on chronically adapted Tibetan population (31, 34, 45). We now add scientific information on the genetic influence of *HMOX2* on the response to acute hypoxia, complementing the study by Yang et al. (35) on adaptation to chronic hypoxia in the Tibetan population, which highlighted the role of rs4786504_*HMOX2* on heme metabolism. Our results showed no significant difference in Hb levels in the two rs4786504_*HMOX2* carrier groups. In male Tibetans, carriers of the C allele have lower Hb levels than T/T carriers (~18 vs. 21 g/dL) (35). In female with lower Hb levels (~16–17 g/dL), there is no difference. These levels (male and female) are all higher than ours, due to chronic exposure to hypoxia with erythropoietin (EPO) secretion. The possible physiological mechanism for lowered Hb levels in male would be based on the activity of HO-2 to degrade heme to biliverdin, CO and Fe^2+^, from a Hb threshold, in order to prevent chronic mountain sickness in populations adapted to high altitude, with the involvement of cofactors (possibly Fe^2+^ status for example). Through further studies, this possible Hb threshold could be defined around 20–21 g/dL, the threshold set for the diagnosis of Monge's disease (46). Hb level is also dependent on tissue oxygen pressure. Subjects with a ventilatory response adapted to hypoxia will have a smaller decrease in tissue oxygen pressure, less EPO secretion, and a lower Hb level. Under chronic hypoxic stress, all carriers of the C/C genotype would therefore be protected from Monge's disease.

The herein presented results are consistent with the role of HO-2 as an “O_2_ sensor” in the human carotid bodies “chemosome” as the first step of hypoxic responses (17) and require confirmation in larger samples. This will also allow to take into account the inter-individual variability and to confirm our results, in particular on the resting HVR, illustrated by the Supplementary Figure 1A. Indeed, the resting HVR is higher than 4 L.min-1.kg-1 for two C/C *HMOX2* carriers. Anticipatory mental stress may have contributed to elevating resting HVR, without impacting the ventilatory response to hypoxia during submaximal exercise (Supplementary Figure 1B) (47). In future studies, the molecular mechanisms responsible for the regulation of ventilation in relation to *HMOX2* and HO-2 activities could additionally be better understood. The functional link between this *HMOX2* polymorphism and individual susceptibility to SHAI needs also to be confirmed in large cohorts, before it can be considered as a predictive marker in a mountain consultation. On the other hand, our results are all the more interesting because new molecular technologies allow us to determine an individual's polymorphism in a single step and in less than an hour, directly from a blood or saliva sample, and without DNA extraction (44, 48, 49). However, in our study, we cannot exclude that other *HMOX2* SNPs may be functional.

The functional polymorphism rs4680_*COMT* modulates the activity of catechol-O-methyl transferase (COMT), a protein whose enzymatic activity results in the degradation of catecholamines. At maximal exercise, the A/A (met/met) mutated genotype' carriers presented lower enzyme activity, increased catecholamines levels and thus higher ANS activity than G/A (met/val) heterozygous or G/G (val/val) ancestral homozygous (39). In acute hypoxia, the ANS and the catecholaminergic system play a direct role in the increases of HR after detection of O_2_ depletion by glomus cells, and also indirectly influence the ventilatory response (50, 51). Indeed, a progressive increase in sympatho-excitation in humans during exposure to high altitude has been found, and this increase has been shown to correlate with the increase in $\overset{\cdot }{V}$E (50). Our results show significant differences between physiological responses to the “hypoxic stress” during the resting phase according to the *COMT* genotypes. Mutated A/A homozygous genotype' carriers have a higher resting HVR, higher measured oxygen saturation and lower calculated difference in oxygen saturation to hypoxia at rest, than the heterozygous G/A carriers. We also observed a trend toward a higher resting cardiac hypoxic response (*p* = 0.156), which needs to be confirmed with a larger sample size. Nevertheless, there is no significant difference related to *COMT* polymorphism during the two sub-maximal exercise phases (normoxic and hypoxic), and, even if there was a significant influence of *HMOX2* and *COMT* polymorphisms individually on resting HVR, and a trend on resting HCR, there was no significant effect of the polymorphism's combination on the calculated parameters HVR, HCR, and DSaO_2_. However, the lack of interaction between *HMOX2* and *COMT* polymorphisms on these responses during hypoxic exercise may be explained by the small sample size of each subgroup.

This study shows no association between the *HMOX2* polymorphism and Hb level in our sample of European sea-level residents. We next compared Hb levels according to detection score for subjects at high risk for SHAI. Tolerance to acute hypoxia appears logically linked to the level of Hb and hematocrit, because Hb is the oxygen carrier, while chronic adaptation require lower increases in Hb levels to prevent chronic mountain sickness (32, 52). Currently, many studies are exploring the relationship between Hb levels and the occurrence of high altitude illnesses in populations living there (33). In acute hypoxia there is rather a focus on high Hb affinity and exercise tolerance (53, 54). In 2009, Richalet et al. present the results between hemoglobin (SaO_2_) association and ventilatory responses in sea-level residents. The subjects with good ventilatory response (HVR+) have higher SaO_2_ levels and lower DSaO_2_ at rest and during exercise in hypoxia compared with subjects with lower ventilatory response (HVR-). In our study, Hb and hematocrit levels were significantly higher in subjects with low risk of SHAI (i.e., score ≤ 5.5) (11, 37). This clinico-physiological score has been validated by a European consensus to detect subjects at high risk of SHAI (13), with a sensitivity of 87.3% and a specificity of 73.1% in subjects with no previous experience at high altitude (37). If our results on Hb and hematocrit levels are confirmed in larger cohorts, these two criteria, easily accessible during a clinical consultation with a questionnaire and a hypoxia stress test, could be considered in the validation of a clinical-physiological and biological score model. In populations living at sea-level, the good responders to hypoxia would be subjects with a good ventilatory response (HVR+), a lower DSa0_2_ to hypoxia and higher Hb and hematocrit levels. In comparison, in populations living at high altitude, chronic hypoxic stress exposes them to chronic mountain sickness (Monge's disease) characterized by high Hb levels, and its complications (46). Subjects adapted to high-altitude living would be those with a good ventilatory response, better SaO_2_, and a controlled increase in Hb and hematocrit (Tibetan populations have acquired these characteristics unlike Andean populations, for example) (34, 35).

In conclusion, this study confirms the relationship between *HMOX2* polymorphism and ventilatory responses to the acute hypoxic exercise test in sea-level European residents likely through variation of individual chemosensitivity. Although these results need to be confirmed in larger samples and in hypobaric rather than normobaric hypoxia, they underline the major role of HO-2 as an “O_2_ sensor”. A better understanding of the genetic factors involved in individual vulnerability to acute hypoxia may improve the prediction of SHAI.

## Data availability statement

The original contributions presented in the study are included in the article/Supplementary material, further inquiries can be directed to the corresponding authors.

## Ethics statement

The studies involving human participants were reviewed and approved by CPP SUD MEDITERRANEE III. The patients/participants provided their written informed consent to participate in this study.

## Author contributions

PF, CD, AM, NK, and MC conceived the experiment. PF, CD, M-CE, ON, VM, WB, BL, and A-PH-H conducted the experiment. PF, CD, FS, DG-M, and MC analyzed the results. PF, CD, FS, ON, AM, NK, DG-M, and MC contributed to the article. All authors approved the submitted version.

## Funding

Funding was provided by the Service de Santé des Armées (French Military Health Service) and Direction générale de l'armement, projet BIOMEDEF MRH1-0725.

## Conflict of interest

The authors declare that the research was conducted in the absence of any commercial or financial relationships that could be construed as a potential conflict of interest.

## Publisher's note

All claims expressed in this article are solely those of the authors and do not necessarily represent those of their affiliated organizations, or those of the publisher, the editors and the reviewers. Any product that may be evaluated in this article, or claim that may be made by its manufacturer, is not guaranteed or endorsed by the publisher.
