# Supplementary material for: The HMOX2 polymorphism contributes to the carotid body chemoreflex in European sea-level residents by regulating hypoxic ventilatory responses
Source: Front Med (Lausanne). 2022 Nov 3;9:1000786. doi: 10.3389/fmed.2022.1000786 (PMC9669423; doi:10.3389/fmed.2022.1000786)
Supplement: Supplementary file 1 [file Data_Sheet_1.pdf]

Supplementary Material

1 Supplementary Figure

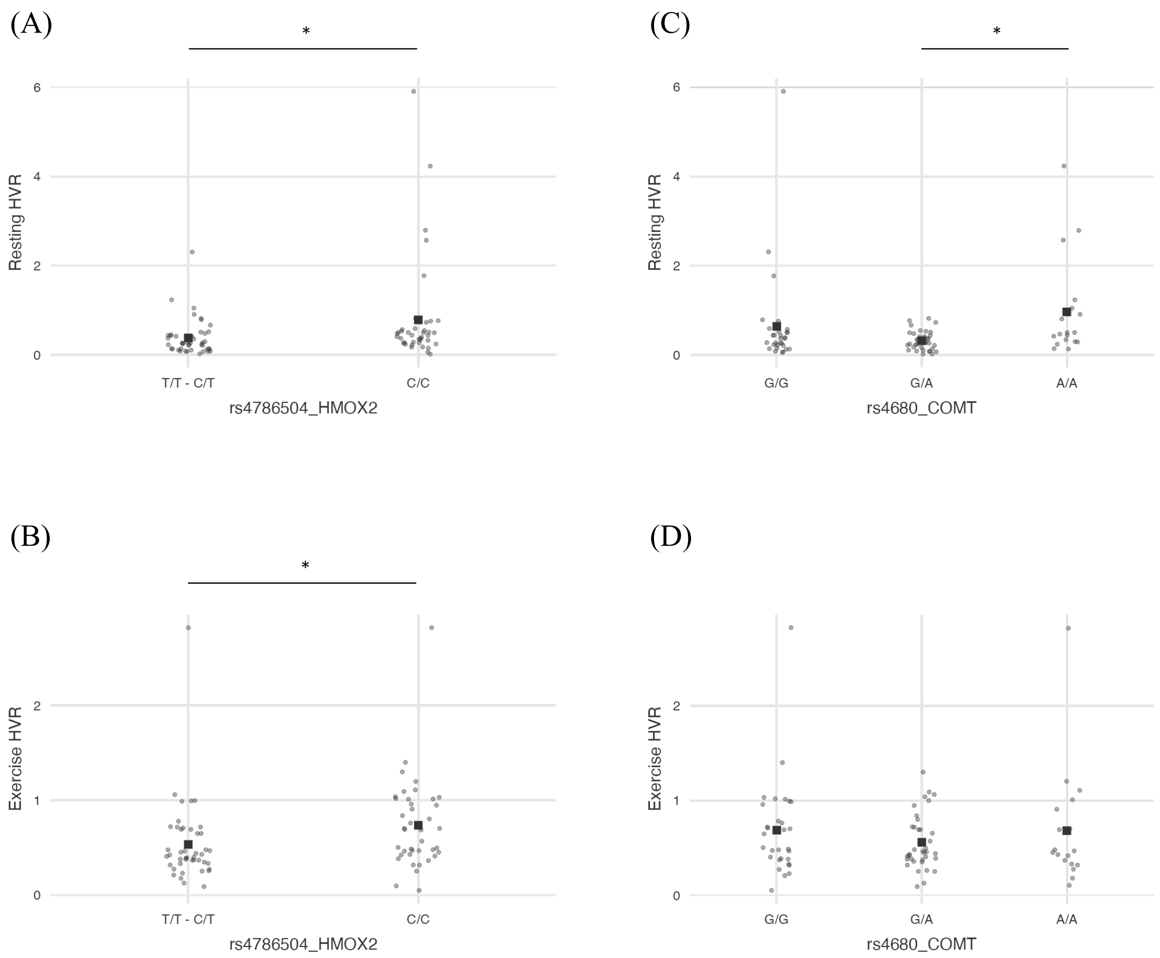

Supplementary Figure 1: Individual values of resting and exercise hypoxic ventilatory responses (HVR, L.min-1.kg-1), as a function of rs4786504_*HMOX2* (A) and (B), and rs4680_*COMT* polymorphisms (C) and (D). The square represents the mean value. * $p < 0.05$
